# Continuing professional development for medical, nursing, and midwifery cadres in Malawi, Tanzania and South Africa: A qualitative evaluation

**DOI:** 10.1371/journal.pone.0186074

**Published:** 2017-10-17

**Authors:** Caryl Feldacker, Jillian Pintye, Sheena Jacob, Michael H. Chung, Lyn Middleton, Jill Iliffe, H. Nina Kim

**Affiliations:** 1 International Training and Education Center for Health (I-TECH), Seattle, WA, United States of America; 2 Department of Global Health, University of Washington, Seattle, WA, United States of America; 3 School of Nursing, University of Washington, Seattle, WA, United States of America; 4 Department of Medicine, University of Washington, Seattle, WA, United States of America; 5 Department of Epidemiology, University of Washington, Seattle, WA, United States of America; 6 Training for Health Equity Network (THEnet), New York, NY, United States of America; 7 School of Health Sciences, College of Health Sciences, University of KwaZulu-Natal, Durban, South Africa; 8 Commonwealth Nurses and Midwives Federation, London, United Kingdom; Universite de Bretagne Occidentale, FRANCE

## Abstract

**Background:**

As innovations in the prevention and treatment of HIV and TB advance, continuing professional development (CPD) of health care workers (HCWs) remains a high priority, particularly in sub-Saharan Africa where dual TB/HIV epidemics are compounded by severe HCW shortages. There is further need to examine CPD programs to identify challenges and effective solutions to strengthen HIV/TB-related CPD.

**Methods:**

Qualitative evaluations in Malawi, Tanzania and South Africa (RSA) were conducted using key informant interviews (KIIs) and focus group discussions (FGDs) in each country to identify barriers and enablers of effective HIV/TB-related CPD. Key stakeholders represented CPD implementers, regulators, and developers. HCWs were purposively sampled from high disease burden districts; each HCW completed brief, semi-structured questionnaires and participated in a FGD. KII and FGD results were combined into key themes spanning across countries using a grounded theory approach.

**Results:**

Fifty-two KIIs were conducted: 17 in Malawi, 19 in Tanzania and 16 in RSA. Eighty-nine HCWs (24 from Malawi, 38 from Tanzania and 27 from RSA) completed questionnaires and participated in FGDs. Primarily, lack of sustainable financial resources and limitations in coordination of CPD result in poor accountability for CPD oversight and reduce CPD quality assurance. Healthcare worker shortages limit CPD opportunities, creating disparities in CPD access. CPD irrelevance and imbalance between HCW-identified CPD needs and current programs reduce enthusiasm for CPD. Facility-level constraints, including poor infrastructure and weak supply chains, restrict implementation of CPD skills and knowledge. Challenges are more severe in rural settings.

**Conclusion:**

To address identified gaps, sustainable funding, strong leadership and collaboration at every level are needed to strengthen CPD regulation and accreditation systems; increase CPD accessibility in the workplace; and create enabling environments for CPD implementation. Together, these improvements may improve TB/HIV CPD quality and patient outcomes.

## Introduction

In sub-Saharan Africa, severe healthcare worker (HCW) shortages threaten efforts to scale up high-quality healthcare, including HIV/TB programs [1–3]. HCW shortages are compounded by weaknesses in HCW pre-service training [4–8], especially in the areas of HIV and TB [9, 10], limiting the preparation of those already in the workforce. Although pre-service education is critical for developing new HCWs, training new workers takes time. To successfully expand HIV and TB treatment in the sub-Saharan Africa region where the epidemic burdens are the highest, efforts must be made to improve the skills and knowledge of those HCWs practicing in the field to more immediately improve care for those in need [11, 12].

Continuing profession development (CPD) aims to ensure that, post-graduation, HCWs have the capacity to practice safely, effectively, and competently to meet changing societal healthcare needs–advances in medicine, revised scopes of practice (task sharing), and emerging health conditions [13]–to meet the ultimate goal of improving quality patient care [14]. HCWs, themselves, also want CPD [4]. In the past decade, renewed commitment from local governments, matched by some donor investment, has led to significant progress in formalization of CPD systems for HCWs in sub-Saharan Africa [15–17]. However, these systems vary considerably in their robustness in this region and the impact of CPD programs remains unknown. This, combined with the general lack of evaluation and research on CPD implementation, leaves both policymakers and program planners with limited evidence to act effectively on behalf of HCWs [18]. There is further need to examine CPD programs at the country-specific level to identify effective solutions for strengthening HIV/TB related CPD programs.

We conducted a study of CPD for nursing, midwifery, and medical cadres in three focus countries: Malawi, Tanzania, and South Africa (RSA). These three countries are notable for their high TB/HIV burdens [19] and, in the case of Malawi and Tanzania, their low provider-to-patient ratios, below the target density of 2.28 HCWs per 1000 people considered adequate for basic healthcare coverage [20]. They differ in their stage of CPD development, regulation, and implementation. A CPD system for nurses, midwives, and doctors is well-established in Malawi; nascent for all cadres in Tanzania; and implemented for doctors in South Africa, with expansion for nurses planned for 2017 (Table 1). Understanding common barriers and actions needed could enable a regional approach to CPD strengthening.

**Table 1:** Overview of CPD regulation for medical, nursing, and midwifery cadres in Malawi, Tanzania, and South Africa as of December 2016*.

Characteristic	Malawi	Tanzania	South Africa
CPD Regulatory Bodies	Nurses and Midwives Council of Malawi (NMCM). Medical Council of Malawi (MCM). MOH Nursing Directorate	Tanzania Nursing and Midwifery Council (TNMC). Medical Council of Tanganyika. Ministry of Health, Community Development, Gender, Elderly and Children (MoHCDGEC)	Health Professions Council of South Africa (HPCSA). HPCSA Medical and Dental Board. South African Nursing Council (SANC). National Department of Health
CPD Guidelines	Guidelines exist for nurses, midwives, and doctors	Draft guidelines were developed for nurses, midwives, and doctors at the end of 2016	Guidelines exist for nurses, midwives, doctors, and various other health professionals including pharmacists, therapists, etc.
Mandatory CPD	Mandatory for license renewal for doctors, nurses, and midwives	MoHCDGEC CPD guidelines make CPD mandatory for all HCWs	Mandatory for license renewal for doctors, nurses, and midwives
CPD Compliance Monitoring	Audit of 5% of registered nurses and midwives; trained CPD facilitators deployed to districts/ facilities to help monitor CPD uptake of practicing nurses and midwives	An audit system for monitoring compliance is yet to be implemented	Audit system in which a certain percentage of HCWs in the registry are sampled and audited at regular intervals
CPD Accreditation	Doctors: The MCM is the main accreditor of CPD providers. Nurses/midwives: No formal CPD accreditation system is yet in place	A formal CPD accreditation system for HCW-related CPD programs has not yet been implemented	SANC is in the process of developing formal guidelines for accreditation; HPCSA delegates the accreditation of CPD activities to providers accredited by professional councils

This study included three aims with a focus on TB/HIV-related content: 1) a review of the CPD policy environment spanning implementation and requirements for professional licensure renewal; 2) thorough mapping of the key stakeholders who regulate and implement in-country CPD activities; and, 3) improved understanding of HCWs’ experiences and preferences with regard to CPD. This paper details our discussion with key stakeholders and HCWs and provides a qualitative, summative examination of barriers and enablers to CPD in Malawi, Tanzania, and RSA.

## Materials and methods

### Ethics

This study was conducted with approvals from the National Health Sciences Research Committee in Malawi, the National Institute for Medical Research in Tanzania, the South African Human Sciences Research Council, and the University of Washington (UW) Human Subjects Division. In all three countries, a similar written informed consent process was approved as part of the ethical review and implemented by the study team. All participants were informed that their participation was voluntary. Participants were assured that their employment would not be affected by their study participation and that their personal information would be kept confidential. Written questionnaires were anonymous. No names were transcribed for the analysis.

### Preparation

A common protocol for study implementation was developed for all three countries, with minimal modification, in consultation with in-country teams of the UW International Training and Education Center for Health (I-TECH). Both focus group discussion (FGD) and key informant interview (KII) guides were developed in English, pre-tested and modified based on pilot testing in English in Malawi and South Africa. In Tanzania, the guides were translated for implementation in Kiswahili. Skilled interviewers were trained in study protocols and pre-tested instruments. The guides for KIIs and FGDs used in RSA are included in supplemental files. Data collection tools were minimally adapted for each country.

### Implementation

This qualitative evaluation is based on gathering rich information from focus groups (FGs) and key informant interviews (KIIs) on preferences, opinions, experiences, and suggestions pertaining to CPD in the participating countries. These qualitative data intend to identify, and provide insights into, the strengths and weaknesses of CPD, thereby evaluating the greater CPD interventions through this respondent-driven method. Unlike quantitative evaluation, this evaluation is not intended to be widely generalizable, but rather to help improve understanding of the diverse and nuanced beliefs expressed by CPD participants and administrators in these three countries. The participant responses in this qualitative evaluation provide more depth and breadth than quantitative results to illuminate challenges and to inform potential solutions to the issues identified.

In each country, the following steps were completed. First, semi-structured key informant interviews were conducted with key stakeholders at the national and regional levels. We used expert and maximum variation sampling to capture as wide a range of perspectives in this domain as we could through a cross-section of different stakeholders; we created lists of potential KIIs in collaboration with in-country staff at I-TECH who were familiar with the local landscape that included “owners” of CPD systems (most often nursing, midwifery, and medical regulatory bodies), organizations that implemented CPD programs, donor organizations that fund CPD programs, pre-service training institutions (e.g., universities, colleges), and professional practice organizations. Interviews lasted approximately one hour. Table 2 includes an overview of the organizations represented through KIIs.

**Table 2:** Key informant organizations by country.

Malawi	Tanzania	South Africa
**Regulatory Bodies**		
**Ministry of Health, HIV Department. Ministry of Health, Nursing Directorate. Medical Council of Malawi. Nurses and Midwives Council of Malawi (NMCM)**	Ministry of Health, Community Development, Gender, Elderly and Children. Ministry of Health, Community Development, Gender, Elderly and Children, Nursing Directorate. Tanzania Nursing and Midwifery Council (TNMC)	National Department of Health, HIV Department. National Department of Health, Nursing Practice. South African Nursing Council (SANC). Health Professions Council of South Africa (HPCSA)
**Professional Associations**		
**National Organisation of Nurses and Midwives of Malawi (NONM). Medical Association of Malawi**	Tanzania National Nurses Association (TANNA). Medical Association of Tanzania. Association of Private Health Facilities in Tanzania (APHFTA)	Democratic Nursing Organization of South Africa (DENOSA). Southern African HIV Clinicians Society
**District Management**		
**District health management team, Lilongwe**	Ilala MMOH*. TB and Leprosy Ilala Municipality	District health management team, Tshwane Municipality
**Facility-Level Stakeholders**		
**Christian Health Association of Malawi (CHAM) Secretariat. Lighthouse Trust HIV Clinic. Baylor International Pediatric AIDS Initiative Center of Excellence—Malawi (BIPAI)**	Buguruni Health Center*. Infectious Disease Center. CTC Amana Hospital*	Life Healthcare Facility, Tshwane Municipality
**Pre-Service Institutions**		
**Malawi College of Health Sciences—Lilongwe Campus. Malawi College of Medicine. Kamuzu College of Nursing. Daeyang Luke College of Nursing, CHAM. Nkhoma College of Nursing and Midwifery, CHAM**	Muhimbili University of Health and Allied Sciences (MUHAS)	University of Pretoria
**CPD Providers**		
**Elizabeth Glaser Pediatric AIDS Foundation (EGPAF)—Malawi. Jhpiego—Malawi**	KNCV Tuberculosis Foundation—Tanzania (KNCV). Ariel Glaser Pediatric AIDS Healthcare Initiative (AGPAHI)—Tanzania. Tanzania Health Promotion Support (THPS). Management and Development for Health (MDH)	Regional Training Centres (RTCs x 2). Foundation for Professional Development (FPD)

*Multiple KIIs were conducted.

Then, in each country, District Managers in high-burden PEPFAR districts were asked to select 40 HCWs to participate in a one-day CPD workshop; one workshop per country. District Managers were asked to select practicing medical, nurse, and midwifery professionals from a cross section of health facilities to represent a variety of HCW cadres and locations. The workshop sessions were interactive and aimed to explore CPD issues with these end-users of CPD. All workshop participants were invited to complete a self-administered survey and to join a focus group of 8–10 HCWs for approximately one hour to discuss their experiences with TB/HIV-focused CPD programming in their respective country. A training with HIV case studies was then provided as an educational offering independent of assessment activities. Participants were also offered room, board, and a locally-appropriate per-diem.

### Data analysis

Verbatim transcription was completed for all recorded KIIs and FGDs. In Tanzania, qualitative results were simultaneously transcribed and translated into English. Qualitative data were entered, coded and analysed as text documents in Atlas.ti 6.0 by two independent, external researchers trained in qualitative methods with content area experience. Analysts followed a hybrid process of both inductive and deductive reasoning to identify a priori and unanticipated themes [21]. First, each document was coded based on predetermined themes covered in the interview guides. Additional themes were identified and coded based on the grounded theory approach [22]–an iterative approach to generating unanticipated thematic concepts and linkages from the data. For codes and themes that were identified by one researcher, but not the other, an iterative process of communication was used to determine the value of the theme and/or code. Moreover, in-country implementation teams reviewed drafts to help affirm neutrality and conformity of the findings to their impressions. All themes presented in the paper were expressed by both FGDs and KIIs; were clear to both researchers or reached through consensus; and were seen as overarching findings from the data. Results from both FGDs and KII were combined and presented as overall challenges and suggestions for future action. Effort was made to reflect different voices from different countries in the selection of clear, concise, illustrative quotes.

## Results

A total of fifty-two key informant interviews (KIIs): 17 in Malawi, 19 in Tanzania and 16 in South Africa were conducted. Three FGDs were conducted in Malawi and RSA; four FGDs were conducted in Tanzania. Eighty-nine HCWs (24 in Malawi, 38 in Tanzania and 27 in RSA) participated in the workshops and comprised the focus groups (FGs). The characteristics of FG participants are detailed in Table 3.

**Table 3:** Focus group participants, by country.

	Malawi	Tanzania	RSA	Total
	(n = 24)	(n = 38)	(n = 27)	(N = 89)
Gender				
Female	18 (75%)	30 (79%)	23 (85%)	71 (80%)
Male	6 (25%)	8 (21%)	4 (15%)	29 (20%)
Age group in years				
20–30	15 (62%)	7 (18%)	5 (19%)	27 (30%)
31–40	4 (17%)	6 (16%)	4 (15%)	14 (16%)
41–50	1 (4%)	16 (42%)	11 (41%)	28 (31%)
>50	4 (17%)	9 (24%)	7 (26%)	20 (23%)
Years of experience				
1–5	12 (50%)	10 (26%)	4 (15%)	26 (29%)
6–10	5 (21%)	4 (11%)	3 (11%)	12 (13%)
11–15	1 (4%)	5 (13%)	4 (15%)	10 (11%)
16–20	1 (4%)	6 (16%)	7 (26%)	14 (16%)
21–25	2 (8%)	2 (5%)	3 (11%)	7 (8%)]
>25	3 (13%)	11 (29%)	6 (22%)	20 (23%)
Current Job/Position				
Physician	0	3 (8%)	1 (4%)	4 (4%)
Registered nurse	6 (25%)	13 (34%)	4 (15%)	23 (26%)
Clinical/medical officer	9 (37%)	8 (21%)	14 (52%)	31 (35%)
Enrolled nurse/NMW	5 (21%)	10 (26%)	1 (4%)	16 (18%)
Nurse assistant	0	1 (3%)	0	1 (1%)
Other	4 (17%)	3 (8%)	7 (26%)	14 (16%)
Location				
Urban	22 (92%)	23 (61%)	13 (50%)	58 (65%)
Rural	2 (8%)	15 (39%)	13 (50%)	30 (34%)
Institution				
Public	24 (100%)	27 (71%)	26 (96%)	77 (87%)
Private	0	5 (13%)	0	5 (6%)
Faith-based	0	5 (13%)	0	5 (6%)
Other	0	1 (3%)	1 (4%)	2 (2%)
Facility				
National/central hospital	10 (42%)	1 (3%)	0	11 (12%)
Regional hospital	0	3 (8%)	2 (7%)	5 (6%)
District hospital	12 (50%)	7 (18%)	9 (33%)	28 (31%)
Urban health center	0	14 (37%)	3 (11%)	17 (19%)
Rural health center	1 (4%)	10 (26%)	5 (19%)	16 (18%)
Other	1 (4%)	3 (8%)	8 (30%)	12 (14%)
Clinical setting				
HIV/ART clinic	6 (26%)	21 (55%)	12 (44%)	39 (44%)
TB clinic	5 (22%)	11 (29%)	3 (11%)	19 (22%)
STI clinic	1 (4%)	0	0	1 (1%)
ANC clinic	0	4 (11%)	2 (7%)	6 (7%)
Other	11 (48%)	2 (5%)	10 (37%)	23 (26%)

NMW, nurse midwife; ART, antiretroviral therapy; TB, tuberculosis; STI, sexually transmitted infection; ANC, antenatal care.

### Challenges to CPD implementation: System level

#### CPD funding

Overall, as noted in almost all key informants (KIs) and focus groups (FGs), donors and both local and international NGOs contribute significantly to CPD; yet more financial resources are needed. At the national level, respondents spoke of the necessity of central financing that guides the development, implementation, and coordination of CPD efforts.

*“It’s time now that all the developmental partners should streamline the support towards building the Ministry of Health’s capacity to coordinate*.*”* [KI-15 Tanzania]

Additional central funding would support more training of HCWs from both public and private sectors and for CPD trainer support to ensure quality facilitation. There is also clear concern about lack of sustainable financial resources at either the individual level (to attend CPD) or the institutional level (to coordinate and/or conduct CPD), making funding a wide barrier to CPD planning, implementation, evaluation, and continuity.

*“The department of health is too dependent on partners*. *When the partners are not there*, *there is no proper training that will take place*.*”* [FG-3 RSA]

#### Gaps in CPD regulation

Across the three countries, respondents noted similarly to this Malawi KI-4 who felt that CPD regulatory bodies “*see themselves as different vertical programs*,” suggesting examination of ways to better reduce redundancy and improve efficiency. NGOs and other partners also develop, implement, and monitor CPD activities, causing some complications with coordination, quality assurance, and regulation.

*“I can’t control what is happening currently because partners come with their funding and their priorities as well*. *But according to us; we don’t like that*, *we want partners to be regulated by our needs and by our priorities; not the other way round*.*”* [KI-15 RSA]

Moreover, improved standardization of CPD requirements should clarify CPD point systems across all implementing partners and regulatory bodies, enabling HCWs to better match their learning needs to their CPD requirements. Lastly, comprehensive and clear guidelines for CPD development, accreditation, implementation, and verification are critical for both implementers and users of CPD.

*“We need to have explicit guidelines of how we going to do this so that you don’t find … inconsistencies in provinces that you are not able to manage*.*”* [KI-10 RSA]

#### Systemic shortages in HCWs affect CPD

The effects of the healthcare worker shortages are profound and felt across countries, more acutely in rural areas. These challenges are compounded by HCW mobility and job changes. Due to lack of human resources, HCWs feel unable to gain access to the CPD they desired or to put their new knowledge into practice. The lack of sufficient staff makes it nearly impossible for some HCWs to leave their posts for CPD or to attend CPD based on need rather than scheduling.

“*I have seen facilities where there is literally one health care professional and one counsellor managing that facility serving a number of villages or communities*, *so obviously pulling out that health care worker [for CPD] whether it’s the counsellor or nurse*, *or whoever*, *it means the system is being crippled*.” [KI-11 RSA]

Even in locations with greater facility-based resources for supplies and equipment, lack of HR curtails CPD implementation, reducing the quality of care.

*“No matter how much you educate or teach someone as much as possible*, *if the work to be done by 10 people is done by only 1 person*, *honestly that will be very difficult”* [FG-3 Tanzania]

#### CPD implementation and quality care impeded by lack of facility-level resources

CPD can only be effective if HCWs have the facility-based resources (equipment, supplies, laboratories, etc.) to enable HCWs to implement new skills and practices. Yet, HCWs across all three countries and across cadres expressed a dire need for additional resources (human, financial, clinical) to enable better practice and ensure quality care.

“*You may be faced with a barrier when you want to implement the issues learned from the trainings*, *because you will have acquired the right knowledge but there is a challenge of medical supplies*, *medicines and other things within your working environment*.*”* [FG-4Tanzania]

At facilities across countries, apparent lack of test kits, medicines, microscopes, broken X-ray machines, etc., severely limit the ability to put CPD into practice, especially in rural settings. Without the tools and supplies to practice quality care skills, CPD efforts are ineffective.

### Challenges to CPD implementation: Implementation level

#### Rural challenges are more profound

Most of the challenges mentioned, including healthcare worker shortages, have more severe consequences in rural areas, a disadvantage frequently reiterated across all three countries. First, access to basic services may be lacking: One KI noted that, “in the rural areas, there is no electricity” [KI-5 RSA]. Rural conditions may also isolate HCWs, leaving them excluded from CPD opportunities.

*“I think for the nurses who are at the district hospitals they are able to acquire CPDs*. *But again for those who are living in other rural areas centres*, *I think it’s too difficult for them to access the CPDs maybe because it has to do with issues of transport and the distance*.*”* [FG-2 Malawi]

Moreover, rural or remote HCWs lack access to the technology from internet access to smart phones; the lack of consistent mobile coverage amplifies these difficulties.

*“Many of them in the rural areas don’t have smart phones… there is also poor phone reception*.*”* [FG-4 Tanzania]

#### Top-down versus HCW-driven selection of CPD topics and trainings

There was a general sentiment that CPD was developed, organized, and delivered centrally without a lot of input from provincial and district levels or HCWs, themselves. Several respondents across countries noted that the one-size-fits-all CPD does not work: CPD needs to be more cadre specific so that people are learning the right content at an appropriate learning level. “*I would recommend that the recipients or beneficiaries, the nurses themselves or clinicians, they should come up with the topics*.” [KI-2 Malawi] Other participants felt that CPD trainings and topics were “donor-driven,” guided by implementing partners, and were not necessarily responsive to local priorities or cultural contexts.

*“Most of the CPDs*, *to my experience*, *I feel*, *its mostly donor funded*, *or donor funding driven…Sometimes*, *the things that the donor doesn’t support*, *the government doesn’t support*, *but they are important*.*”*[KI-17 Tanzania]

Additionally, although the HCWs recognize that both TB and HIV merit CPD focus, many respondents from all three countries noted that TB takes a consistent backseat to HIV-related care, reducing efforts at both TB control and TB/HIV integration.

*“Especially TB*, *[it’s] like it’s being side lined; we have given much weight on HIV but TB it is like nobody is talking about it but it is there*.*”* [KI-10 Tanzania]

Relatedly, as HCWs may have little voice in CPD related decisions, some staff complain about the lack of flexibility to meet their CPD requirements, resulting in HCWs taking CPD for convenience not interest.

### Challenges to CPD implementation: Individual level

#### Lack of self-motivation

HCWs, themselves, note attitudes that challenge CPD implementation. One Tanzanian respondent (KI-16) suggested that it is critical for “*the staff to realize "that CPD is their personal responsibility*” while another in Malawi (KI-9) reflected, “*learning has to be driven by the learner!*” Although the need for self-motivation was well recognized, one RSA participant (KI-5) noted that they, “*are not geared to that culture of appreciating that in-service it is something that actually strengthens you as a person*.” Some HCWs noted that their motivation is diminished by the way in which HCWs are selected for CPD: the same HCWs are sent repeatedly, training does not match the attendee’s job, or participant selection criteria does not meet the CPD provider’s expectations. “*We are not given the same opportunities and there is favoritism in terms of who goes to where*.” [KI-6 RSA]

#### Money for attendance

Although closely linked to the area of funding, the issue of individually-allocated per diems for training attendance plagues Malawi and other countries in the region, although less so in South Africa. The practice of payment for attendance existed for decades: many believe that “*you have just gone to “eat” the money wherever you were*” [FG-2 Tanzania]. Although the idea that CPD is attractive only for related financial gain is waning, there remains an association of training with monetary incentives. In Malawi, when “*they don’t get any allowance … they have been boycotting the sessions*” [KI-12 Malawi]. Without per diems, a clear challenge is a general lack of motivation for CPD among HCWs, mostly in Malawi but in Tanzania as well. And the money issue appears to permeate further than individual recipients. Some comments suggested that HCWs who received a CPD allowance should share that money with the supervisor who selected them or with colleagues during information dissemination.

#### Country-specific challenges

There were a few minor issues that set the countries apart. In Malawi, where CPD system is more mature, funding was the predominant issue, ranging from issues in lack of central resources to the challenge presented by the per diem expectation. In Tanzania, respondents also noted the need to include private practitioners in the CPD regulations. Tanzanian respondents noted that providers from private facilities and community-based organizations should be considered in CPD activities as they have needs that are largely ignored. Lastly, in South Africa, issues of HCW labor union involvement in CPD-related decisions and the concerns about legal issues surrounding quality care provision were distinct to this context. Also, as CPD for doctors is more solidified there than for nurses, there is a clear call for additional standardization and regulation for nurses as their CPD becomes compulsory.

#### Suggestions for CPD improvement

Within the key informant interviews and focus group discussions, rich conversations emerged around recommendations to improve CPD, in general, rather than focused specifically on TB/HIV-related CPD. These suggestions, taken from the conversations, illustrate a few broad considerations for regional initiatives to increase the positive impact and potential for sustainability of CPD, including those efforts aimed at TB/HIV CPD.

#### Ensure appropriate facility resources for CPD implementation

Across KIIs and FGDs, almost all participants expressed a dire need for additional human resources and medical supplies to enable better practice and ensure the application of acquired CPD skills.

“*I will give an example of the health centres, they will call a TB-HIV training, where they will learn about the Gene Xpert and how to diagnose TB in HIV infected patients but if they have never seen these machines and have never worked on them, they wouldn't know what to order, how to do it, and how to just carry out the whole procedure…so it's very difficult for them to implement that information when you don’t have the resources on the ground*” [FG-1 Malawi]

Without the enabling environments to practice quality care skills, CPD efforts are ineffective, wasting resources and decreasing healthcare quality.

#### Implement CPD and follow-up mentoring in the workplace

A clear recommendation was to take CPD sessions to the workplace since “mentorship is much better than being in the class, and as we know the mentor goes to your place of work” [KI-11 Malawi]. Workplace CPD was noted, specifically, to improve access for rural areas, reducing barriers such as limited time for CPD, reduced CPD access, poor facility resources, transportation, distance, HCW shortages, and connectivity. On-site mentorship was also considered beneficial to tailor content to the reality of the HCWs in their facility:

“*I think the method of mentorship is good where they can visit us at our workplace for issues of supervision*, *to check if we are doing the correct things*, *to see if the things taught in class are done as required”* [FG-1 Tanzania]

#### Provide more quality assurance for CPD implementation

Accreditation systems for CPD would provide a platform for improving the quality of CPD trainings. The need for more monitoring and evaluation along the entire CPD spectrum was an overarching theme noted by many participants across countries. Relatedly, facilitators should be selected based on their clinical skills, well trained to teach to the level of each cadre, and rotated around districts to better equalize trainings across providers.

“*You can conduct a training but if the trainer is not very competent …you will end up with someone who is not even knowledgeable because of the trainers*. *But we have very few trainers; I wish they would be trained more and more*.*”* [KI-4Tanzania]

Tracking HCW CPD participation could also provide an additional tool and quality assurance mechanism to best match CPD offerings to CPD needs.

#### Increase access to technology

The lack of computers and internet access extends from facilities into the institutions and government offices, reducing access to information and online CPD opportunities. Expanding eLearning may increase CPD options, especially for rural sites; however, this will require broad-based multilateral investments in both the infrastructure (broadband internet, electricity) and hardware (computers/ tablets/ smartphones) as well as training experts to improve computer literacy of HCWs. Although it appears that technology access is improving, and many respondents mentioned that such access is not universal.

“*Not all villages have access to electricity, and not many health facilities have been connected to electricity supply, in addition to the lack of devices such as laptops, smartphone to be able to learn in that style.*” [FG-2 Tanzania]

#### Find CPD champions at all levels

Effort is needed to promote strong CPD leadership at national, regional, and facility levels. Respondents across countries noted that good leadership increases the potential for positive CPD impact.

“*One of the contributing factors [for CPD to be successful] could be leadership at that particular institution because if the leader takes it positively*, *know exactly what this CPD is all about*, *then definitely is going to encourage the update of knowledge and skills for the subordinates*”[Malawi KI-10].

Engaging high-capacity CPD champions may also promote stronger HCW performance and help foster a positive culture of CPD that relies less on distracting external motivators, such as per diem for training attendance, and more on internal motivators, such as lifelong learning and the desire to provide high quality HIV care.

## Discussion

The results from this qualitative evaluation present several key barriers and enablers to TB/HIV CPD in the region. The themes that emerged in our evaluation of the state of health sector CPD in these countries have been outlined in a variety of policy statements and reviews on HCW education over the last decade [23–30] and have called attention to the systemic weaknesses that impact CPD. CPD has historically been implemented as a response to shortcomings in HCW numbers, distribution, and training; however, CPD is not a panacea, particularly if it takes the form of fragmented training along disease-specific agendas that are implemented by external partners and designed around short-term goals and funding cycles. The usual top-down approach to selecting participants for such programs has also contributed to ineffectual CPD, tailored neither to the scope of work nor to the realities of clinical practice. CPD cannot in itself address the strains on the health sector due to HCW shortages, maldistribution of workers, or the inefficient, poor-quality care that can result from a weak health care delivery system.

The pervasive nature of barriers to developing quality HCWs across countries and cadres merits critical attention. Lack of quality CPD may reduce HCW motivation, job retention and job satisfaction [4, 31, 32]. Without increased opportunities for CPD, including better distribution [33], HCWs may become further demotivated and stymied by restricted career growth [34]. If access improves, it appears that better tracking of CPD attendance by topic and cadre would improve CPD equity and distribution as well as verification for relicensing purposes. To complement CPD system strengthening, HCWs must have the tools and supplies that support their training as facility-based constraints reduce HCW motivation and diminish quality care [34–36]. Furthermore, as monetary incentives for HCW training may distort HCW motivation in more resource-limited settings [37–39], consideration for reducing or removing individual-level incentives for CPD attendance in the region is warranted along with decentralizing these activities to the workplace.

Financial and personnel resources are clearly needed to bolster TB/HIV CPD in the region. Technical assistance to regulatory bodies, including relevant sections of the ministries of health and professional medical and nursing/midwifery councils could strengthen their skills to plan, implement, monitor, and evaluate CPD programs. In line with WHO encouragement to ensure that all clinical cadres benefit from accreditation and regulation processes [40], CPD should be made mandatory for all professional cadres and linked to professional license or registration to create accountability, oversight, and fairness in CPD access. Clear national guidelines for CPD implementation must be also available and accessible by both providers and recipients of CPD to help lessen confusion about CPD requirements.

There are several limitations of our study worth noting. First, the largely qualitative study was intended to be both small-scale and rapidly implemented to explore HCW experiences and expert opinions, reducing the scope of the activity. Second, in each country, key staff interviewed were largely selected by convenience sample and fewer participants than expected attended the workshops, potentially reducing the representativeness of the findings. Caution should be employed when extrapolating beyond the included HCWs and countries. Furthermore, as the CPD systems in Tanzania, Malawi, and South Africa vary widely and our primary focus was on TB/HIV-related content, our recommendations and results may not be generalizable to all cadres and other topics within these countries. Despite these limitations, we believe that the study provides key insights into the implementation and effects of CPD in these three countries, providing lessons that are worthy of consideration in the region overall.

## Conclusions

CPD is but one critical component in a spectrum of interventions needed to strengthen healthcare service delivery in the sub-Saharan African region. Ultimately, sustained improvements in this arena will require a coordinated, long-term, and holistic effort that addresses all aspects of health care strengthening—wages, working conditions, health education, accreditation/regulation, and information systems—to create enabling environments for learning and to work towards the transformative model of health education proposed by the WHO [41]. This more comprehensive effort would include 1) financial support to both pre-service training and to improvements in working conditions for existing health workers, 2) reinforcing regulation mechanisms and processes linked to both accreditation of health training institutions and individual health workers; and 3) delivering CPD in an integrated manner that reflects the broader clinical responsibilities of these HCWs and actual disease contexts of the population served.

## Supporting information

S1 File Key informant interview and focus group guide. (DOCX)
