# Supplementary material for: Continuing professional development for medical, nursing, and midwifery cadres in Malawi, Tanzania and South Africa: A qualitative evaluation
Source: PLoS One. 2017 Oct 17;12(10):e0186074. doi: 10.1371/journal.pone.0186074 (PMC5645103; doi:10.1371/journal.pone.0186074)
Supplement: S1 File — (DOCX) [file pone.0186074.s001.docx]

Key Informant Interview Guide

South Africa Landscape Analysis of CPD

*Semistructured Key Informant Interview (KII) Guide*

**Total Interview Time:** 45 min–1 hr.

**You will need:** KII interview guide, a KII consent form, a fully-charged tape recorder, and blank paper for notetaking.

Section I: Introduction

*“Hello, our names are __________________________________.*

We are working on a project for the Ministry of Health and the University of Washington based in Seattle, Washington, in the United States. The Ministry of Health would like you to help us understand more about your experience with HIV- and TB-related continuing professional development (CPD) programs for health care workers, specifically nursing, midwifery, and medical cadres, in South Africa. The information you provide will be very useful to help inform future CPD programs for health care workers and ultimately to help identify ways to strengthen HIV and TB services delivery in the country.

*We would like to thank you for agreeing to participate in this interview. You have been asked to join us here because you are a key stakeholder who helps regulate, plan, or implement CPD activities for HCWs. We will ask you questions about your experience with CPD programs including your input regarding the strengths of CPD activities to date and how CPD programs can be improved.*

*We would like to review this consent form for the interview with you now. (REVIEW KII CONSENT FORM WITH PARTICIPANT.) You can choose not to discuss any topic and you are free to leave at any time. Are there any questions?*

*Do you agree to participate? (ASK PARTICIPANT TO SIGN AND DATE THE CONSENT FORM IF THEY AGREE TO PARTICIPATE.)*

*We would like to remind you that there are no right or wrong answers in this interview. We encourage you to be as honest as possible and to speak freely with us today.”*

Section II: Interview Questions

1. From your perspective, what is the overall purpose of CPD?

|  |
| --- |
|  |

1. How have you been involved with HIV- or TB-related CPD programs to date?

|  |
| --- |
|  |

1. Who are the other key stakeholders in CPD programs? How do you collaborate/work/communicate with these stakeholders? [Prompts: email correspondence, conference calls, individual 1:1 meetings, technical working group meetings, etc.?]

|  |
| --- |
|  |
|  |

1. How do these stakeholders work together to plan/implement HIV- and TB-focused CPD activities? [Prompt: Are there any challenges to collaboration?]

|  |
| --- |
|  |
|  |

1. What do you see/consider the HIV- and TB-related content [knowledge or information] gaps in CPD for HCWs in the country? (Examples: paediatric HIV clinical management, viral load testing, managing 2nd and 3rd line ART, etc.) [Prompt: Are there any other knowledge gaps you can think of?]

|  |
| --- |
|  |

1. Aside from traditional classroom training for CPD, what additional CPD interventions might help HCWs perform better at HIV clinical facilities?

**Probe:** onsite clinical mentoring support

**Probe:** how could technology help? For example, having access to computers, tablets, smartphones with the latest clinical guidelines, or HIV-/TB-related training modules?

**Probe:** integrating hands-on practicum training during CPD trainings

**Probe:** anything else?

|  |
| --- |
|  |

1. How is the quality of the existing CPD program ensured?

|  |
| --- |

1. Has the impact/benefit of CPD programs been measured? And if so, how?

|  |
| --- |
|  |

1. How can lay/community health care workers be included in CPD programs?

|  |
| --- |
|  |

1. What are the overall strengths of the CPD program?
2. What do you think have been the key factors that have led to these successes?

|  |
| --- |
|  |

1. What are the main challenges/limitations of the CPD program?
2. Are these challenges the same in urban and rural settings? Tell me more…
3. Are HCWs who are required to task share or task shift receiving enough training to do their jobs well? Tell me more…

|  |
| --- |
|  |

1. What recommendations do you have for strengthening the overall CPD program? [For example, “You mentioned that X has been a major challenge. How do you think it could be addressed?”]

|  |
| --- |
|  |

Section III: Conclusion

Thank you for taking time out of your busy schedule to provide us with this valuable input today! Your contributions will go a long way with helping to strengthen CPD for HCWs across the country.

Focus Group Discussion Guide

South Africa CPD Needs Assessment

*Focus Group Discussion (FGD) Guide*

**Total Interview Time:** 1 hr.

**You will need:** One FGD facilitator, one note taker, a FGD guide, FGD consent forms (for every FGD participant), a fully charged tape recorder, and **Focus Group Field Note Paper** for note taking.

Section I: Introduction

“Hello, our names are __________________________________.

We are working on a project for the Ministry of Health and the University of Washington based in Seattle, Washington, in the United States. The Ministry of Health would like you to help us understand more about your experience with HIV- and TB-related continuing professional development (CPD) programs for health care workers, specifically nursing, midwifery, and medical cadres, in South Africa . The information you provide will be very useful to help inform future CPD programs for health care workers and ultimately help to identify ways to strengthen HIV and TB services delivery in the country.

We would like to thank you for agreeing to participate in this interview. You have been asked to join us here because you are a health care worker who participates in CPD programs in order to strengthen your skills to provide HIV and/or TB care at facilities across the country. We will ask you questions about your experience with CPD programs including your input regarding the strengths of CPD activities to date and how CPD programs can be improved.

We would like to review this consent form for the interview with you now. (REVIEW FGD CONSENT FORM WITH PARTICIPANTS.)

You can choose not to discuss any topic and you are free to leave at any time. Are there any questions? Do you agree to participate? (ASK EACH PARTICIPANT TO SIGN AND DATE THE CONSENT FORM IF THEY AGREE TO PARTICIPATE.)

We would like to remind you that there are no right or wrong answers in this interview. We encourage you to be as honest as possible and to speak freely with us today. We also ask that you provide each member of this group with the opportunity to voice their opinions for each question. We want to respect everyone’s contributions in this discussion forum.”

Section II: Interview Questions

“Before we begin recording, could you please introduce yourselves? Then, once we begin recording, please do not use names in the discussion.”

[COMPLETE INTRODUCTIONS, REMIND ALL NOT TO USE NAMES, AND BEGIN RECORDING]

Interview Questions

1. From your perspective, what is the overall purpose of CPD?
2. Why do you participate in HIV/TB related CPD trainings? [Tell me more about how it benefits you.]
3. Do CPD trainings address your specific HIV/TB specific knowledge/skills gaps (or meet your specific learning objectives for HIV/TB CPD)? If so how? If not, why not?
4. Aside from traditional classroom training for CPD, what other types of CPD might help HCWs perform HIV/TB skills better at clinical facilities?

**Probe:** onsite clinical mentoring support

**Probe:** how could technology help? For example, having access to computers, tablets, smartphones with the latest clinical guidelines, or HIV-/TB-related training modules?

**Probe:** integrating hands-on practicum training during CPD trainings

**Probe:** anything else?

1. How would you describe the quality of the HIV/TB CPD trainings you attended? [Probes: Tell me more. How else could the quality be strengthened?)
2. What helps you implement new knowledge/skills gained from a CPD training once you return to your workplace?
3. What prevents you from implementing new knowledge/skills gained from a CPD training once you return to your workplace?
4. What do you think are the main challenges/limitations to HIV-/TB-specific CPD programs?
5. Are these challenges the same in urban and rural settings?
6. What recommendations do you have for strengthening HIV-/TB-specific CPD programs? For example, “You mentioned that X has been a major challenge. How do you think it could be addressed?”]

Section III: Conclusion

Thank you for taking time out of your busy schedule to provide us with this valuable input today! Your contributions will go a long way with helping to strengthen CPD for HCWs across the country.
